# Capecitabine monotherapy as first-line treatment in advanced HER2-normal breast cancer – a nationwide, retrospective study

**DOI:** 10.2340/1651-226X.2024.38886

**Published:** 2024-06-23

**Authors:** Alan Celik, Tobias Berg, Magnus Gibson, Maj-Britt Jensen, Iben Kümler, Saskia Eßer-Naumann, Erik H. Jakobsen, Ann Knoop, Dorte Nielsen

**Affiliations:** a Danish Breast Cancer Group, Rigshospitalet, Copenhagen University Hospital, Copenhagen, Denmark; b Department of Clinical Oncology, Rigshospitalet, Copenhagen University Hospital, Copenhagen, Denmark; c Department of Oncology, Herlev Hospital, Copenhagen University Hospital, Herlev, Denmark; d Zealand University Hospital, Department of Oncology, Naestved, Denmark; e Hospital of Southern Jutland, Department of Oncology, Sonderborg, Denmark; f Department of Clinical Medicine, University of Copenhagen, Copenhagen Denmark

**Keywords:** Breast cancer, advanced breast cancer, HER2-normal, Capecitabine, chemotherapy, overall survival, progression free survival

## Abstract

**Background and purpose:**

Capecitabine can be used as first-line treatment for advanced breast cancer. However, real-world data on efficacy of capecitabine in this setting is sparse. The purpose of the study is to evaluate outcomes of patients with Human Epidermal Growth Factor Receptor (HER2)-normal advanced breast cancer treated with capecitabine monotherapy as first-line treatment.

**Material and Methods:**

The study utilized the Danish Breast Cancer Group (DBCG) database and was conducted retrospectively across all Danish oncology departments. Inclusion criteria were female patients, with HER2-normal advanced breast cancer treated with capecitabine monotherapy as the first-line treatment from 2010 to 2020. The primary endpoints were overall survival (OS) and progression-free survival (PFS).

**Results:**

A total of 494 patients were included. Median OS was 16.4 months (95% confidence interval [CI]: 14.5–18.0), and median PFS was 6.0 months (95% CI: 5.3–6.7). Patients with estrogen receptor (ER)-positive disease had significantly longer OS (median: 22.8 vs. 10.5 months, p < 0.001) and PFS (median: 7.4 vs. 4.9 months, p = 0.003), when compared to ER-negative patients. Stratifying by age, patients under 45 years displayed a median PFS of 4.1 months, while those aged 45–70 years and over 70 years had median PFS of 5.7 and 7.2 months, respectively (p = 0.01).

**Interpretation:**

In this nationwide study, the efficacy of capecitabine as a first-line treatment for HER2-normal advanced breast cancer is consistent with other, mainly retrospective, studies. However, when assessed against contemporary and newer treatments, its effectiveness appears inferior to alternative chemotherapies or targeted therapies.

## Introduction

Every year, approximately 4,500 Danish women are diagnosed with breast cancer, with around 5% of these women presenting with primary metastatic disease (de novo), and a further 10%–30% experiencing a systemic relapse within 10 years of their initial breast cancer diagnosis [1, 2].

Individuals diagnosed with breast cancer can be categorized into subtypes based on their hormonal and molecular characteristics. These categories include estrogen receptor (ER)-positive, Human Epidermal Growth Factor Receptor 2 (HER2)-positive, and triple-negative breast cancer (TNBC), with TNBC distinguished by the absence of both ER expression, progesterone receptor expression, and HER2 amplification. In a study involving 22,000 patients with metastatic breast cancer, the distribution of these subtypes was found to be 62%, 18%, and 13%, respectively [3]. Treatment options for advanced breast cancer rely on chemotherapy, targeted therapy (e.g. HER2-directed therapy, Cyclin-dependent Kinase 4/6 (CDK4/6) inhibitors, antibody-drug conjugates), endocrine therapy, immunotherapy, or combinations of these therapies [4, 5].

Capecitabine, an oral pro-drug of fluoropyrimidine, was approved by the European Medicines Agency (EMA) in 2001 for the treatment of advanced breast cancer [6, 7]. Initially, it was approved as a monotherapy option after failure of prior taxane and anthracycline or as combination therapy with docetaxel after prior anthracycline therapy failure. Clinical trials, including prospective randomized phase II/III trials, have demonstrated the antitumour activity of capecitabine as a first-line treatment for advanced breast cancer. It has been studied as monotherapy and in combination with other agents [8–11]. First-line capecitabine monotherapy has shown a favourable safety profile, with no significant myelosuppression or alopecia [12, 13]. Capecitabine is available in tablet form and can be administered long-term without the cumulative toxicity associated with other chemotherapy agents.

While capecitabine is not considered the standard-of-care first-line treatment for any subtype according to the guidelines of the American Society of Clinical Oncology (ASCO) or the European Society for Medical Oncology (ESMO), it is mentioned as one of several options for first-line treatment in patients with ER-positive disease in specific circumstances outlined by both guidelines. These circumstances may include viscerally dominant disease (ASCO) or visceral crisis (ESMO) [4, 5, 14–16]. The standard first-line treatment for patients with metastatic ER-positive, HER2-normal breast cancer (and no imminent organ failure) is combination therapy with CDK4/6 inhibitors and endocrine therapy. Results from the PEARL study indicated no significant enhancements in progression-free survival for patients with aromatase inhibitor-resistant metastatic ER-positive, HER2-normal breast cancer. However, the CDK4/6-inhibitor, palbociclib, combined with endocrine therapy demonstrated a superior safety profile and enhanced quality of life compared to capecitabine treatment [17]. Consequently, international guidelines do not endorse the utilization of capecitabine as the standard-of-care first-line treatment. Nevertheless, it remains one of the preferred treatment options used in Denmark.

Although capecitabine was approved over 20 years ago based on clinical trials, recent knowledge about capecitabine derives from its use as a control arm in studies evaluating newer chemotherapies and biological agents, either alone or in combination with capecitabine [17, 18].

The current literature lacks real-world evidence on first-line capecitabine as treatment for patients with HER2-normal advanced breast cancer. Our study aims to provide a description of outcomes of patients who received capecitabine as first-line treatment for their advanced breast cancer, utilizing a nationwide database.

## Material and methods

### Study design

This is a nationwide, retrospective, observational real-world evidence study involving all Danish departments of oncology.

### Patient selection

All women at least 18 years old who initiated treatment with capecitabine monotherapy as first-line treatment for HER2-normal metastatic or locally advanced breast cancer between January 1, 2010, and December 31, 2020, were eligible for inclusion. Patients were excluded if they had received treatment with another agent prior to or concurrently with capecitabine in the first-line.

### Data source

The primary data source for this study was the DBCG’s database. The database includes data on diagnosis, patient demographics, tumour features, treatments, and follow-up. Both the organisation of the DBCG and its database have been described in detail before [19, 20]. Information on vital status was acquired from the Civil Registration System with complete information until September 1, 2023.

### Endpoints

The primary endpoints of this study were overall survival (OS) and progression free survival (PFS).

### Measures

De novo metastatic breast cancer refers to patients who are diagnosed with confirmed metastatic disease either at the time of their initial diagnosis or within a maximum of 90 days following surgery. The index date was defined as the date of diagnosis of metastatic/locally advanced disease. ER- and HER2-status were determined from metastatic site biopsies or primary tumour data when metastatic site information was unavailable. In Denmark, patients diagnosed with tumours that are ER-positive (≥10%) or have 1–9% ER positivity and exhibit the luminal A/B subtype (e.g. as determined by Prediction Analysis of Microarray 50 [PAM50]), are deemed to be ER-positive. HER2 status was evaluated via immunohistochemistry (IHC), where scores of 3+ were classified as HER2-positive, while tumours scoring 0 or 1+ were categorized as HER2-normal. For tumours scoring 2+ on IHC, Fluorescence in situ Hybridization (FISH) analysis was performed. Tumours found to be amplified during FISH were defined as HER2-positive. Disease progression was assessed according to RECIST 1.1 criteria, using radiological and clinical examinations conducted by the treating departments every 8–12 weeks [21].

### Statistical analysis

PFS was defined as the time from index date to either progression, death from any cause, or end of clinical follow-up; whichever occurred first. OS was defined as the time between the index date and death from any cause or end of clinical follow-up; whichever occurred first. OS and PFS were estimated using the Kaplan–Meier method. Subgroup analysis of OS and PFS was done by disease presentation (de novo metastatic vs. recurrent breast cancer), ER-status (positive vs. negative), age (< 45 vs. 45–70 vs. > 70), and adjuvant chemotherapy (yes vs. no). Formal statistical test was conducted for subgroup-analyses of OS and PFS using Log-Rank method. Estimated potential follow-up was computed using the reverse Kaplan–Meier method, wherein the roles of event status and censored data were inverted. Follow-up time was subsequently reported in medians, along with confidence intervals (CIs). Testing was done by Wilcoxon rank sum test, Pearson’s Chi-squared test, or Fisher’s exact test when subgroups were compared by patient characteristics.

## Results

### Patient population

**Figure 1:**
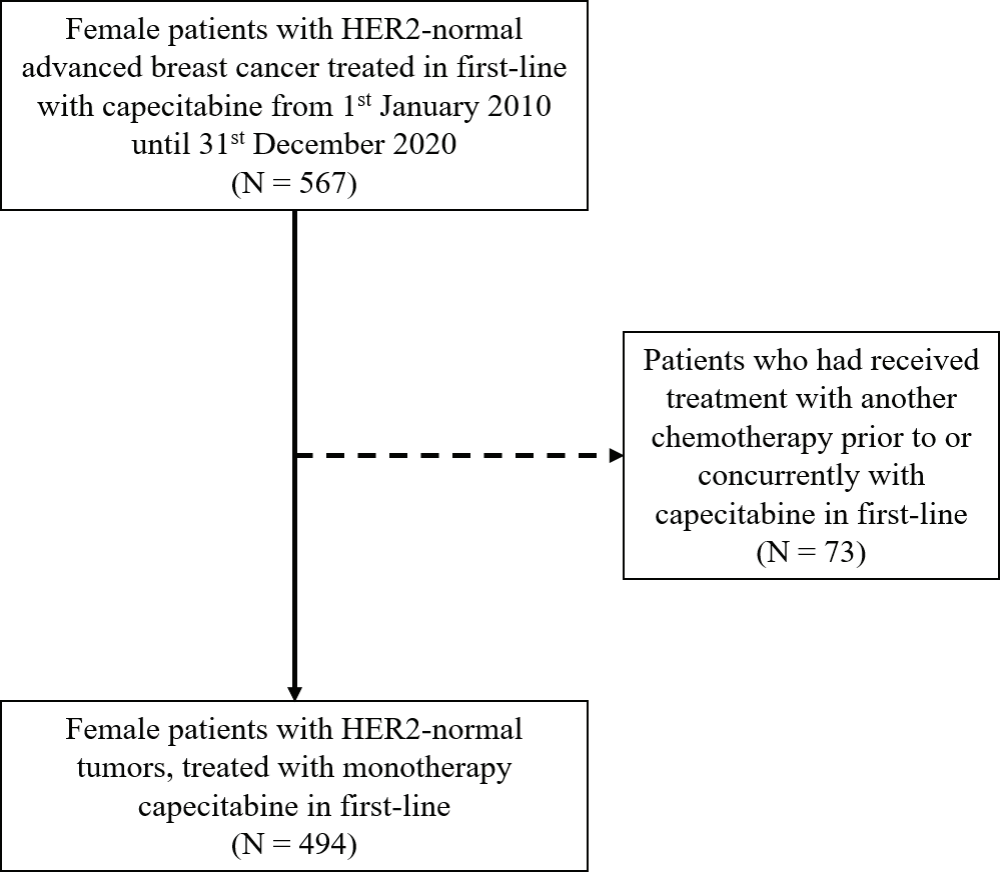
Flowchart of patient selection. HER2: human epidermal growth factor receptor 2.

From January 1, 2010, to December 31, 2020, 494 patients were identified, starting capecitabine monotherapy as first-line treatment for HER2-normal advanced breast cancer (Figure 1).

Patient characteristics are presented by ER-status in Table 1 and by disease presentation in Table 2.

**Table 1:** Patient characteristics stratified by estrogen receptor status.

Characteristic	Overall, *N* = 494	ER-positive, *N* = 252	ER-negative, *N* = 242	*p*-value
**Age** ^1^	64 (52, 73)	63 (51, 72)	65 (53, 74)	0.01
<45	55 (11%)	30 (12%)	25 (10%)	
≥45 & ≤70	265 (54%)	140 (56%)	125 (52%)	
>70	174 (35%)	82 (33%)	92 (38%)	
**Disease presentation**				0.009
Recurrent BC	448 (91%)	237 (94%)	211 (87%)	
De novo mBC	46 (9.3%)	15 (6.0%)	31 (13%)	
**Metastatic site**				
Visceral	319 (65%)	175 (69%)	144 (60%)	0.02
CNS metastases	36 (7.3%)	16 (6.3%)	20 (8.3%)	0.40
**Number of sites**				0.02
1–2	270 (55%)	151 (60%)	119 (49%)	
≥3	224 (45%)	101 (40%)	123 (51%)	

1 Median (IQR); *n* (%).

ER: estrogen receptor; BC: breast cancer; mBC: metastatic breast cancer; CNS: central nervous system.

**Table 2:** Patient characteristics stratified by disease presentation.

Characteristic	Overall, *N* = 494	Recurrent, *N* = 448	De novo, *N* = 46	*p*-value
**Age^1^**	64 (52, 73)	62 (52, 72)	74 (68, 79)	<0.001
<45	55 (11%)	51 (11%)	4 (8.7%)	
≥45 & ≤70	265 (54%)	254 (57%)	11 (24%)	
>70	174 (35%)	143 (32%)	31 (67%)	
**Metastatic site**				
Visceral	319 (65%)	291 (65%)	28 (61%)	0.60
CNS-metastases	36 (7.3%)	34 (7.6%)	2 (4.3%)	0.60
**Number of sites**				0.06
1–2	270 (55%)	251 (56%)	19 (41%)	
≥3	224 (45%)	197 (44%)	27 (59%)	
**Adjuvant treatment**				
Chemotherapy^2^		260 (58%)		
*Combination anthracycline and taxane*		218 (84%)^3^		
*Anthracycline alone*		14 (5%)^3^		
*Taxane alone*		19 (7%)^3^		
Endocrine therapy^2^		184 (78%)^4^		

1 Median (IQR); *n* (%)

2 No chemotherapy: *n* = 112 (25%), unknown chemotherapy: *n* = 76 (17%). No endocrine therapy: *n* = 16 (7%)^5^, unknown endocrine therapy: *n* = 37 (16%)^5^.

3 Percentage of patients who received chemotherapy (*N* = 260).

4 Percentage of patients who are ER-positive (*N* = 237).

ER: estrogen receptor; CNS: central nervous system.

Patients with ER-positive disease constituted 51% of the cohort. Patients with ER-negative disease were more likely to be older (*p* = 0.01) and present with de novo metastatic breast cancer (*p* = 0.009). Visceral metastases were more prevalent in patients with ER-positive disease (69% vs. 60%) (*p* = 0.02). However, patients with ER-positive disease had lesser tumour burden with 40% having three or more metastatic sites at baseline compared to 51% of patients with ER-negative disease (*p* = 0.02). No significant differences were found according to presence of CNS-metastases (*p* = 0.40) (Table 1).

A total of 448 (91%) patients presented with recurrent disease while 46 (9%) patients had de novo metastatic breast cancer. Patients with de novo metastatic breast cancer were significantly older than patients with recurrent breast cancer (median age 74 vs. 62, *p* < 0.001) (Table 2).

Of the 448 patient who presented with recurrent breast cancer, 260 (58%) had received (neo)adjuvant treatment with chemotherapy. Of these, 251 (97%) patients received anthracycline and/or a taxane. In total, 218 (84%) patients received a combination of anthracycline and taxane, 14 (5%) patients received anthracycline alone, and 19 (7%) received taxane alone. Seventy-eight percent of the patients with recurrent ER-positive disease had received adjuvant endocrine therapy (Table 2).

### Outcomes

**Figure 2:**
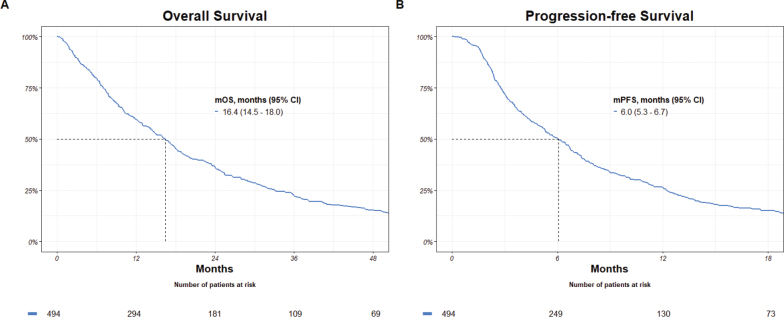
Overall and progression-free survival. mOS: median overall survival; mPFS: median progression-free survival; CI: confidence interval.

During an estimated median potential follow-up for OS of 91.4 months, 458 events were observed. Median OS was 16.4 months (95% CI: 14.5 – 18.0). The estimated median potential follow-up for PFS was 80.7 months and 479 events were observed. Median PFS was 6.0 months (95% CI: 5.3 – 6.7) (Figure 2).

#### Subgroup analyses: Overall survival

**Figure 3:**
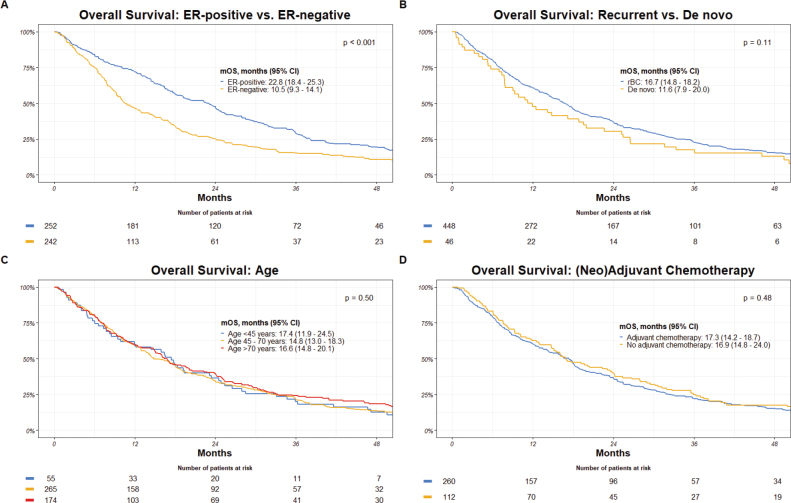
Overall survival by: (A) ER-receptor-status, (B) disease presentation, (C) age, and (D) (neo)adjuvant chemotherapy. mOS: median overall survival; CI: confidence interval; ER: estrogen receptor; rBC: recurrent breast cancer.

Patients with ER-positive breast cancer displayed a significantly extended OS with a median of 22.8 months, compared to 10.5 months for those with ER-negative disease (*p* < 0.001). Differences, though not statistically significant, also emerged in the context of disease presentation, with recurrent breast cancer patients demonstrating a median OS of 16.7 months and de novo metastatic breast cancer patients showing a median OS of 11.6 months (*p* = 0.11). Age-stratified analyses revealed little variation, as individuals under 45 years displayed a median OS of 17.4 months, while those aged 45–70 years and over 70 years had median OS of 14.8 and 16.6 months, respectively (*p* = 0.50). Furthermore, the impact of (neo)adjuvant chemotherapy on OS was explored. Patients who received (neo)adjuvant chemotherapy had a median OS of 17.3 months, similar to the median OS of 16.9 months for those who did not receive (neo)adjuvant chemotherapy (*p* = 0.48) (Figure 3).

### Subgroup analyses: Progression free survival

**Figure 4:**
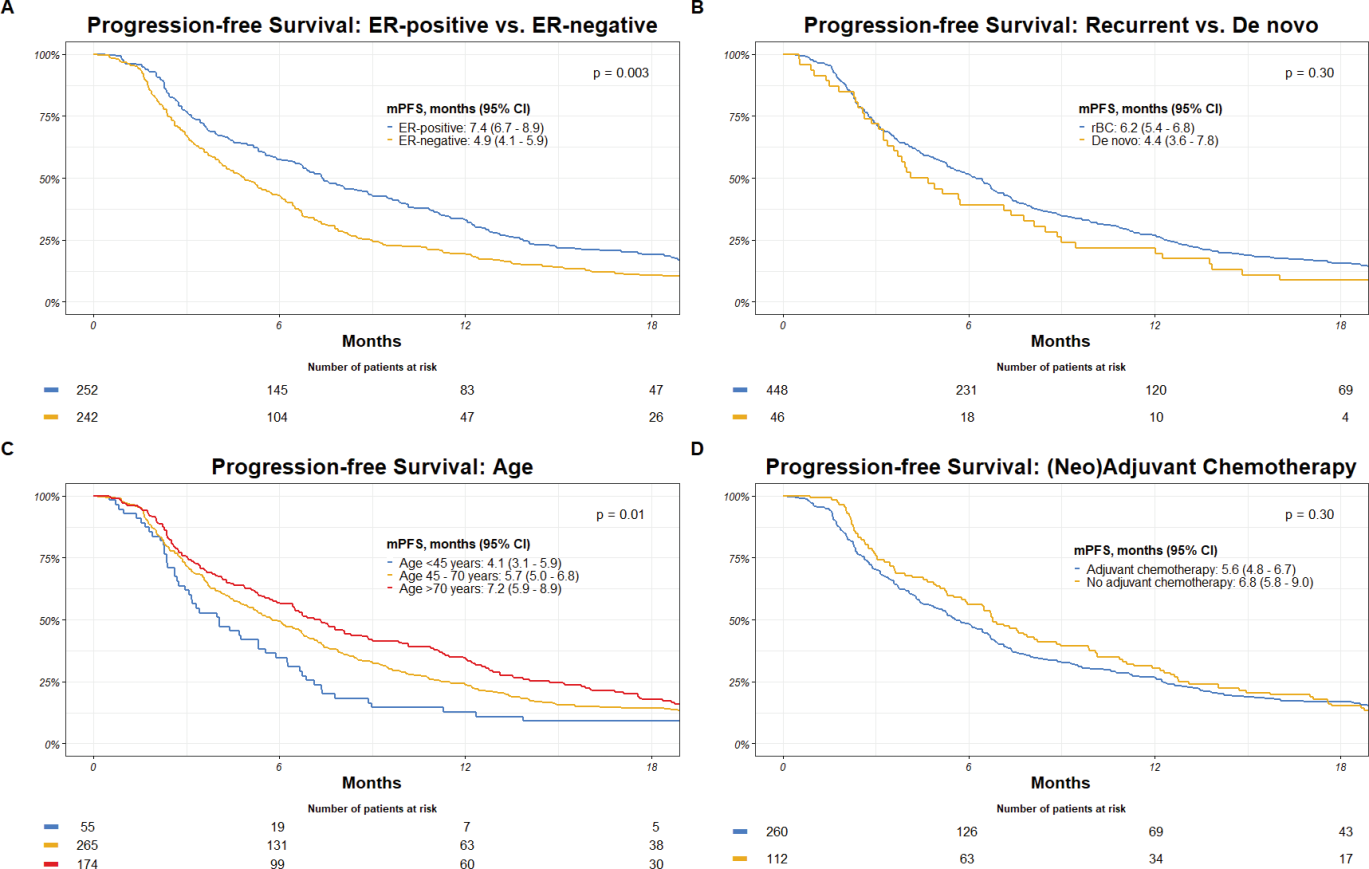
Progression-free survival by: (A) ER-receptor-status, (B) disease presentation, (C) age, and (D) (neo)adjuvant chemotherapy. mPFS: median progression free survival; CI: confidence interval; ER: estrogen receptor; rBC: recurrent breast cancer

Patients with ER-positive disease demonstrated a median PFS of 7.4 months, which was significantly longer than the median PFS of 4.9 months observed in those with ER-negative breast cancer (*p* = 0.003). Again, a non-significant difference was present concerning disease presentation, with recurrent breast cancer patients exhibiting a median PFS of 6.2 months and de novo metastatic breast cancer patients having a median PFS of 4.4 months (*p* = 0.30). Stratifying patients by age, individuals under 45 years displayed a median PFS of 4.1 months, while those aged 45–70 years and over 70 years had median PFS of 5.7 and 7.2 months, respectively (*p* = 0.01). Patients who underwent (neo)adjuvant chemotherapy exhibited a median PFS of 5.6 months, which was not significantly different from the median PFS of 6.8 months observed in those who did not receive (neo)adjuvant chemotherapy (*p* = 0.30) (Figure 4).

## Discussion

Information on real-world efficacy of capecitabine as a first-line treatment in advanced breast cancer is sparse. To our knowledge, this is the first nationwide study evaluating the real-world efficacy of capecitabine monotherapy as a first-line treatment for advanced breast cancer. Our study found a median OS of 16.4 months (95% CI, 14.5 – 18.0), and a median PFS of 6.0 months (95% CI, 5.3 – 6.7).

Previous studies on capecitabine as monotherapy have shown median PFS from 3.1 to 7.9 months [8, 12, 13, 22–24]. Differences in treatment lines may explain shorter median PFS in some studies. Comparable real-world data are scarce in regard to first-line capecitabine and are mainly retrospective in nature [23, 24]. Some effort has been made to produce comparable evidence in reviews as well [8, 12, 13, 22]. Table 3 presents a comparison between the current study and the other six referenced studies across type of study, line(s) of treatment and percentage of patient previously treated with anthracyclines and/or taxanes.

**Table 3:** Overview of studies evaluating treatment with capecitabine in treatment of HER-non-amplified advanced breast cancer.

Study (year)	Type of study	Number of patients	Line(s) of treatment	Median PFS/TTP	Median OS	Percentage pre-treated with anthracyclines and/or taxanes
Blum et al. (2012)	Pooled analysis of RCTs	*n* = 268	1^st^ (2^nd^ line data not used)	4.9 months	21.9 months	57%
Alsaloumi et al. (2020)	Meta-analysis (RCTs)	*n* = 3,257	Multiple (not specified)	3.1 – 5.4 months	13.1 months	100%
Oostendorp et al. (2011)	Systematic review	*n* = 1,174	Multiple (not specified)	4.2 / *3.9* months	13.5 months	100%
Thijssen et al. (2021)	Real-world study, retrospective, observational, single centre	*n* = 506	1^st^ to 5^th^	*6.4* months	13.3 months	98.8%
Babacan et al. (2015)	Real-world study, retrospective, observational, single centre	*n* = 109	1^st^	7.0 months	30.0 months	69%
O’Shaughnessy et al. (2012)	Review of RCTs	*n* = 958	1^st^ (primarily)	6.0 – 7.9 months	18.6 – 29.4 months	Variable
*This study*	*Real-world study, retrospective, observational, nationwide*	*n = 494*	*1* ^st^	*6.1 months*	*16.4 months*	*51%*

PFS: progression-free survival; OS: overall survival; TTP: time to progression; RCT: randomized controlled trials.

A significant difference in OS and PFS was demonstrated for patients with ER-positive and ER-negative advanced breast cancer. In line with this, other studies evaluating capecitabine treatment in advanced breast cancer display a significant difference in outcomes between HER2-normal/ER-positive and triple-negative patients [8, 22, 23, 25].

When solely assessing the ER-positive patient group, the median PFS and median OS from our study compared significantly unfavourably, especially in studies evaluating CDK4/6-inhibitors and/or endocrine therapy as first-line treatment for ER-positive advanced breast cancer [3, 17, 26–34]. In comparison, a recent Danish real-world study evaluating the CDK4/6-inhibitor palbociclib in a first-line setting showed a median PFS of 24.3 months and median OS of 51.7 months [34]. Despite several alternative treatment modalities, such as CDK4/6-inhibitors (introduced in 2017 in Danish practice), having surfaced over the years for ER-positive advanced breast cancer patients, there is still a use of capecitabine as first-line therapy. An average of approximately 20 ER-positive patients were included in our study annually from 2017. This indicates a lack of shift to newer, and maybe more effective treatments, in Danish clinical practice. A possible explanation for the inferior PFS and OS found in our study may be attributed to the nature of real-world studies versus randomized clinical trials where patients must meet strict inclusion criteria for meaningful participation and often present with few, if any, comorbidities. When comparing results from our study with first-line chemotherapy for advanced breast cancer, clinical reasoning behind choosing capecitabine as the first-line treatment must be considered. Thus, patients treated with capecitabine in the first-line may have experienced visceral crisis or have been unable to tolerate treatment with other, intravenous, chemotherapies [5, 15]. It is fair to assume such a population would have a worse prognosis when compared to a general population of ER-positive advanced breast cancer patients. During the study period, chemotherapy has primarily been recommended for patients with rapidly progressing disease, particularly in cases of visceral crisis in Denmark [35]. However, recent studies suggest that patients with performance status scores of 0–2 should be recommended antihormonal therapy with a CDK4/6-inhibitor instead of chemotherapy in cases of visceral crisis [36, 37]. Supplementary survival analyses were conducted for subgroups with and without visceral metastases, which showed significantly lower PFS and OS for the subgroup with visceral metastases (*p* < 0.001) (Supplementary Table 1).

Regarding TNBC, median PFS and OS for this study corroborated with results from a Danish real-world study as well as two reviews on TNBC treatments [38–40]. When outcomes for the ER-negative population are compared to studies evaluating PD-1/PD-L1 inhibitors, such as KEYNOTE-522, IMpassion130 and IMpassion131 with median PFS ranging from 5.7 to 7.5 months and median OS ranging from 17.2 to 22.1 months, the outcomes of the present study fall short [41–43].

Our study showed no significant difference in OS and PFS concerning disease presentation. Prior research indicates that patients with HER2-positive de novo disease exhibit significantly improved PFS and OS compared to those with recurrent disease, as evidenced by findings from a real-world Danish population [44]. Furthermore, among ER-positive patients treated with CDK4/6 inhibitors, superior PFS and OS outcomes are observed in patients with de novo metastatic disease [45]. Conversely, for TNBC, outcomes vary. A Danish real-world study highlights the fact that TNBC patients with de novo disease experience worse PFS and OS compared to those with recurrent disease [38]. However, when chemotherapy is combined with immunotherapy for the same TNBC patient group, those with de novo disease demonstrate extended PFS and OS [46]. Nevertheless, it is important to interpret our current cohort’s OS and PFS differences cautiously due to the limited sample size of the de novo group (*n* = 46).

Patients who had received (neo)adjuvant chemotherapy (218 patients received a combination of anthracycline and taxane, 14 patients received an anthracycline alone and 19 received taxane alone) did not have a significantly different PFS or OS when compared to patients being chemotherapy-naïve. This lack of significant difference in outcomes is unexpected when considering previous literature that examines the impact of exposure to adjuvant chemotherapy on the efficacy of first-line chemotherapy for advanced breast cancer [47, 48]. Patients differed in terms of PFS between age group, with the youngest patients having the shortest PFS (*p* = 0.01). No significant difference was found in OS (*p* = 0.50). The underlying reasons for this discrepancy in PFS could stem from several factors. One plausible explanation is that younger patients may present with more aggressive disease profiles as also seen in early breast cancer populations [49–53].

This study holds certain strengths and limitations. This study utilized the DBCG national database, and all patients known to initiate capecitabine as the first-line treatment for their advanced disease were included, minimizing biases related to socioeconomic factors and geographic variations. Furthermore, the study had a long inclusion and follow-up period, which enhances the robustness of outcome assessments. The study lacks information regarding performance status, comorbidities, objective response rates, DPYD genetic testing, and data concerning the safety of treatment. Furthermore, the study had a restricted sample size within the de novo subgroup (*n* = 46), rendering the establishment of robust scientific evidence challenging.

## Conclusion

Capecitabine monotherapy treatment is often preferred due to relatively manageable side effects and possibility for peroral administration. However, the outcomes assessed in terms of PFS and OS are not impressive, which should be stated when involving the patient in shared decision. When outcomes were compared with contemporary and newer treatments, the efficacy as a first-line treatment, in both ER-positive and ER-negative populations might be limited.

## Author contributions

A.C.: Conceptualization, data curation, formal analysis, investigation, methodology, writing – original draft, writing – review & editing.

T.B.: Conceptualization, data curation, investigation, methodology, supervision, visualization, writing – review & editing.

M.G.: Data curation, formal analysis, writing – original draft.

M.J.: Conceptualization, data curation, methodology, supervision, writing – review & editing.

I.K.: Investigation, writing – review & editing.

S.E.: Investigation, writing – review & editing.

E.J.: Investigation, writing – review & editing.

A.K.: Conceptualization, investigation, methodology, project administration, supervision, validation, visualization, writing – review & editing.

D.N.: Conceptualization, investigation, methodology, project administration, supervision, validation, visualization, writing – review & editing.

## Disclosure of interest

A.C.: Unrestricted research grant from Danish Cancer Society.

T.B.: Institutional grants from: Pfizer, Astra Zeneca, Novartis, Samsung Bioepis, Seattle Genetics, Merck, Eli Lilly and Daiichi Sankyo. Advisory board: Novartis. Travel: Daiichi Sankyo.

M.G.: Unrestricted research grant from Danish Cancer Society.

M.J.: Advisory board, Novartis.

I.K.: None.

S.E.: Institutional grants from Novartis og Merck.

E.J.: None.

A.K.: Institutional grants from Pfizer, AstraZeneca, Merck, Eli Lilly, Seattle Genetics, Roche, Novartis. Personal grants from Astra Zeneca (travel + advisory board), MSD (travel), Daiichi Sankyo (advisory Board), Novartis (advisory Board), Seagen (advisory Board), Gilead (advisory Board).

D.N.: None

## Data availability statement

All data are stored in the DBCG database. The dataset can be made available to qualified researchers through application to the Danish Breast Cancer Group. Please contact dbcg.rigshospitalet@regionh.dk.

## Ethics declarations & trial registry information

The study was approved by the Danish Breast Cancer Group’s oncological committee. The study was also registered and approved by the Capital Regions research overview (P-2021-656).

## Supplementary Material

Capecitabine monotherapy as first-line treatment in advanced HER2-normal breast cancer – a nationwide, retrospective study
