# Supplementary material for: Capecitabine monotherapy as first-line treatment in advanced HER2-normal breast cancer – a nationwide, retrospective study
Source: Acta Oncol. 2024 Jun 23;63:38886. doi: 10.2340/1651-226X.2024.38886 (PMC11332473; doi:10.2340/1651-226X.2024.38886)
Supplement: Capecitabine monotherapy as first-line treatment in advanced HER2-normal breast cancer – a nationwide, retrospective study [file AO-63-38886-s1.pdf]

Supplementary material has been published as submitted. It has not been copyedited, or typeset by Acta Oncologica

Supplementary Table 1: Median progression free and overall survival stratified by metastatic sites.

Group	Median PFS	p-value[1]	Median OS	p-value[1]
**Metastatic site**				
Non-visceral	6.9 months (95% CI, 6.1 – 8.9)	<0.001	20.1 months (95% CI, 18.2 – 25.6)	<0.001
Visceral	5.3 months (95% CI, 4.2 – 6.5)		13.0 months (95% CI, 10.3 – 16.1)	

[1]Log-rank test
